# Rapid induction of p21^WAF1^ but delayed down-regulation of Cdc25A in the TGF-**β**-induced cell cycle arrest of gastric carcinoma cells

**DOI:** 10.1038/sj.bjc.6690478

**Published:** 1999-06

**Authors:** S H Kang, Y-J Bang, H-S Jong, J Y Seo, N K Kim, S-J Kim

**Affiliations:** Cancer Research Center, and Department of Internal Medicine, Seoul National University College of Medicine, Seoul, 110-799, Korea; Laboratory of Cell Regulation and Carcinogenesis, National Cancer Institute, Bethesda, MD, 20892-5055, USA

**Keywords:** TGF-β, cell cycle arrest, cdk inhibitors, p21^WAF1^, Cdc25A, gastric cancer cells

## Abstract

Transforming growth factor-β (TGF-β) is a multifunctional polypeptide that inhibits cellular proliferation in most epithelial cells. cdk4 and several cyclin-dependent kinase (cdk) inhibitors (p15^INK4B^, p21^WAF1/Cip1^ and p27^Kip1^) have been implicated in the TGF-β-induced cell cycle arrest. More recently, down-regulation of Cdc25A, a cdk activator, was additionally suggested as a mechanism underlying growth inhibition by TGF-β. The existence of diverse cellular mediators of TGF-β, however, raises the question of whether their involvement might occur in a redundant manner or coordinately in a certain cell type. Using two TGF-β-sensitive gastric carcinoma cell lines (SNU-16 and -620), we addressed the contributory roles of several cdk inhibitors, and of cdk4 and Cdc25A, in TGF-β-induced cell cycle arrest by comparing their temporal expression pattern in response to TGF-β. Among the cdk inhibitors examined, p21 mRNA was most rapidly (in less than 1 h) and prominently induced by TGF-β. In contrast, p15 mRNA was more slowly induced than p21 in SNU-620 cells, and not expressed in SNU-16 cells harbouring homozygous deletion of p15. Western blotting results confirmed the rapid increase of p21, while opposite patterns of p27 expression were observed in the two cell lines. The down-regulation of Cdc25A mRNA occurred, but was more delayed than that of p15 or p21. Until G1 arrest was established, changes in the protein levels of both Cdc25A and cdk4 were marginal. Co-immunoprecipitation with anti-cdk4 antibody showed that induced p21 associates with cdk4 and that its kinase activity is reduced by TGF-β, which kinetically correlates closely with G1 arrest following TGF-β treatment of both cell lines. These results suggest that in certain human epithelial cells, p21 may play an early role in TGF-β-induced cell cycle arrest, and its cooperation with other cdk inhibitors is different depending on cell type. Delayed down-regulation of Cdc25A and cdk4 may contribute to cell adaptation to the quiescent state in the two gastric carcinoma cell lines studied. © 1999 Cancer Research Campaign


*British Journal of Cancer* (1999) **80**(8), 1144–1149
© 1999 Cancer Research Campaign
Article no. bjoc.1999.0478

### Rapid induction of p21$^{WAF1}$ but delayed down-regulation of Cdc25A in the TGF-β-induced cell cycle arrest of gastric carcinoma cells

**SH Kang[1], Y-J Bang[1,2], H-S Jong[1], JY Seo[1], NK Kim[1,2] and S-J Kim[3]**

[1]Cancer Research Center and [2]Department of Internal Medicine, Seoul National University College of Medicine, Seoul 110-799, Korea; [3]Laboratory of Cell Regulation and Carcinogenesis, National Cancer Institute, NIH, Bethesda, MD 20892-5055, USA

**Summary** Transforming growth factor-β (TGF-β) is a multifunctional polypeptide that inhibits cellular proliferation in most epithelial cells. cdk4 and several cyclin-dependent kinase (cdk) inhibitors (p15$^{INK4B}$, p21$^{WAF1/Cip1}$ and p27$^{Kip1}$) have been implicated in the TGF-β-induced cell cycle arrest. More recently, down-regulation of Cdc25A, a cdk activator, was additionally suggested as a mechanism underlying growth inhibition by TGF-β. The existence of diverse cellular mediators of TGF-β, however, raises the question of whether their involvement might occur in a redundant manner or coordinately in a certain cell type. Using two TGF-β-sensitive gastric carcinoma cell lines (SNU-16 and -620), we addressed the contributory roles of several cdk inhibitors, and of cdk4 and Cdc25A, in TGF-β-induced cell cycle arrest by comparing their temporal expression pattern in response to TGF-β. Among the cdk inhibitors examined, p21 mRNA was most rapidly (in less than 1 h) and prominently induced by TGF-β. In contrast, p15 mRNA was more slowly induced than p21 in SNU-620 cells, and not expressed in SNU-16 cells harbouring homozygous deletion of p15. Western blotting results confirmed the rapid increase of p21, while opposite patterns of p27 expression were observed in the two cell lines. The down-regulation of Cdc25A mRNA occurred, but was more delayed than that of p15 or p21. Until G1 arrest was established, changes in the protein levels of both Cdc25A and cdk4 were marginal. Co-immunoprecipitation with anti-cdk4 antibody showed that induced p21 associates with cdk4 and that its kinase activity is reduced by TGF-β, which kinetically correlates closely with G1 arrest following TGF-β treatment of both cell lines. These results suggest that in certain human epithelial cells, p21 may play an early role in TGF-β-induced cell cycle arrest, and its cooperation with other cdk inhibitors is different depending on cell type. Delayed down-regulation of Cdc25A and cdk4 may contribute to cell adaptation to the quiescent state in the two gastric carcinoma cell lines studied.

**Keywords:** TGF-β; cell cycle arrest; cdk inhibitors; p21$^{WAF1}$; Cdc25A; gastric cancer cells

Transforming growth factor-β (TGF-β) is a multifunctional homodimeric polypeptide which acts as a negative growth regulator in most epithelial and endothelial cell types (Massague, 1990). Loss of TGF-β responsiveness is likely to result in deregulated cellular growth in vivo, and is thus thought to be an important step in the development of various epithelial tumours (Filmus and Kerbel, 1993; Markowitz and Roberts, 1996). While it is firmly established that the growth inhibitory effect of TGF-β is mediated by a heterodimeric receptor complex comprising two distinctly related transmembrane serine/threonine kinases (Wrana et al, 1994), the intracellular components underlying this effect still require further definition despite extensive documentation of the involvement of numerous molecules (Polyak, 1996).

It has been suggested that positive growth regulators such as c-*myc*, G1 cyclins and cdk4 are molecular targets of TGF-β (Pietenpol et al, 1990; Ewen et al, 1993; Geng and Weinberg, 1993). The discovery of two families of cyclin-dependent kinase (cdk) inhibitors provided novel insights into the negative regulation of cell growth (Nasmyth and Hunt, 1993). Since then, other cdk inhibitors, namely p27$^{Kip1}$, p15$^{INK4B}$ and p21$^{WAF1/Cip1}$ have been listed as potential cellular mediators in TGF-β-induced cell cycle arrest (Hannon and Beach, 1994; Polyak et al, 1994*a*, 1994*b*; Datto et al, 1995; Li et al, 1995). The induction or redistribution of those molecules effectively suppressed their specific G1 cdk–-cyclin targets.

With regard to this multiplicity of cell cycle regulatory molecules, efforts have been made to unify the actual cellular mechanism responsible for the anti-mitogenic effect of TGF-β. On the basis of the results with two normal epithelial cell lines (e.g. Mv1Lu and HaCaT), a model in which the action of cdk inhibitors is compromised has been suggested (Reynisdottir et al, 1995). It argues that TGF-β initially induces p15, which then cooperates with a Kip/Cip cdk inhibitor (p27 or p21), suppressing both major cell cycle engines (cdk4 and cdk2), and leading to G1 arrest. More recently, Cdc25A, a cdk activator, has been shown to be rapidly repressed by TGF-β in human mammary epithelial cells, and has been suggested as an alternative mechanism of inhibiting cdk4 in p15-deficient but TGF-β-sensitive cells (Iavarone and Massague, 1997).

Although there is now a better understanding of the potential involvement of these various cell cycle regulators, the results of these studies have been derived from a limited set of cell lines. With regard to the effects of TGF-β on the cyclin–cdk systems, the

*Received 14 July 1998*
*Revised 6 January 1999*
*Accepted 7 January 1999*

*Correspondence to:* Y-J Bang, Department of Internal Medicine, Seoul National University Hospital, 28 Yongon-Dong Chongno-Ku, Seoul 110-799, Korea interpretation of most findings has sometimes been hampered by inconsistent results in different cell systems (Alexander and Moses, 1995). Whether these cdk inhibitors and cdk activators play a similar role in all cell types is therefore an important question.

In the present study, we examined the response of cdk inhibitors, cdk4 and Cdc25A after TGF-β treatment of gastric carcinoma cell lines. p21 was found to be the most prominent CDK inhibitor, being rapidly induced by TGF-β. The response of p15, Cdc25A and cdk4, however, was delayed or marginal. SNU-16 cells, which has lost both copies of p15 gene, appears capable of mediating the TGF-β effect in the absence of p15 via p21. In both TGF-β-sensitive cell lines, moreover, p21 activation closely matched the kinetics of G1 arrest. These results provide further evidence for an initiative role of p21 in the TGF-β-induced antiproliferative effect on some epithelial cells.

### MATERIALS AND METHODS

### Cell lines

Four gastric carcinoma cell lines [SNU-1, -5, -16 and -620] were kindly provided by Dr Jae-Gahb Park of the Department of Surgery, Seoul National University Hospital. They had been established from primary tumours or malignant ascites of gastric cancer patients, either directly or after heterotransplantation in athymic nude mice (Park et al, 1990, 1997). Cells were cultured in RPMI-1640 medium supplemented with 10% fetal bovine serum.

### Growth inhibition by TGF-β

Actively growing asynchronous cells were plated into 96-well plates at a density of $1 \times 10^4$ cells per well and treated with various concentrations of up to 200 pM porcine TGF-β1 (R&D) for 72 h. To compare cellular confluence between individual groups, colorimetric (3-(4,5-dimethylthiazol-2-yl)-2,5-diphenyl-tetrazolium bromide (MTT)) assay was performed (Carmichael et al, 1987). This may reflect the sensitivity of cells to the growth inhibitory effect of TGF-β.

### Cell cycle distribution analysis

To monitor TGF-β-derived changes in the G1 population with time, crude DNA content of an individual group was measured. After incubation in the presence of TGF-β for various lengths of time, TGF-β-sensitive cells were suspended in 1 ml solution of 0.1% sodium citrate and 0.1% Triton X-100 containing 50 μg of propidium iodide per 1 ml, and then treated with 1 μg $ml^{-1}$ of RNAase for 30 min at room temperature. DNA fluorescence was measured using a FACStar flow cytometer (Becton Dickinson, San Jose, CA, USA).

### Northern blot analysis

Total RNA was isolated from cells according to the acid guanidinium isothiocyanate–phenol–chloroform method (Chomczynski and Sacchi, 1987). For more sensitive visualization of p15 and Cdc25A transcripts, poly-adenosine (poly-A) RNA was further extracted using Oligotex mRNA midi kit (QIAGEN GmbH, Hilden, Germany) according to the manufacturer's instruction. Fifteen micrograms of total RNA or 2 μg of poly-A RNA was electrophoresed on a 1% agarose gel containing 0.66 M formaldehyde, before being transferred to a nylon membrane (Nytran; Schleicher and Schuell, Keene, NH, USA), and cross-linked using a UV Stratalinker (Stratagene). Blots were hybridized in 50% formamide, 5 × saline–sodium phosphate–EDTA, 0.5% sodium dodecyl sulphate (SDS), 5× Denhardt's solution at 42°C for 20 h. $^{32}P$-labelled cDNA probes were prepared by using human p15, p16, p21, p27, Cdc25A, c-*myc* and β-actin cDNA fragments as templates.

### Western blot analysis

Cells were washed in phosphate-buffered saline (PBS), and lysed in TNES buffer (50 mM Tris pH 7.5, 1% NP40, 2 mM EDTA, 10 mM sodium chloride, 20 μg $ml^{-1}$ aprotinin, 20 μg $ml^{-1}$ leupeptin, 1 mM phenylmethylsulphonyl fluoride) on ice for 30 min One hundred micrograms of whole cell lysates were resolved on 12% SDS-polyacrylamide gel, transferred onto nitrocellulose membrane (Bio-Rad) by electroblotting and probed with anti-p21 monoclonal antibody (Oncogene Science), anti-p27 (Transduction Laboratory, Lexington, KY, USA), anti-cdk4 polyclonal antibody (Santa Cruz) or anti-Cdc25A polyclonal antibody (Santa Cruz). The blots were developed by using the ECL kit (Amersham, Arlington Heights, IL, USA).

### Immunoprecipitation and cdk4 kinase assay

After 12 h of TGF-β treatment, SNU-620 cells were lysed in TNES buffer containing 1 mM sodium vanadate (TNESV buffer). Immunoprecipitation was performed using 1 μg of polyclonal anti-cdk4 antibody with 300 μg of cell lysates and was followed by Western blot analysis for p21. To assess changes in cdk4 kinase activity, anti-cdk4 immunoprecipitates were prepared after incubations for various periods with TGF-β, washed several times with TNESV buffer, then twice with kinase buffer (20 mM Tris, pH 8.0, 10 mM magnesium chloride, 1 mM EGTA). Twenty microlitrss kinase buffer containing 0.1 μg purified GST-Rb (Kaelin et al, 1991) and 5 μCi [$\gamma^{32}P$]ATP were then added. The reactions were incubated for 10 min at 30°C, separated on denaturing gels and detected by autoradiography.

### RESULTS

### Growth inhibition of gastric carcinoma cell lines by TGF-β

To assess TGF-β sensitivity and the dose–response of four gastric carcinoma cell lines, we measured cellular confluence by MTT assay after 72 h culture at various concentrations of TGF-β. Two of four cell lines (SNU-16 and -620) were very sensitive to TGF-β, while the others (SNU-1 and -5) were resistant (Figure 1). The half maximal inhibitory concentration ($IC_{50}$ values) of both sensitive cells was < 6.25 pM. These growth profiles were consistent with previous results obtained by DNA synthesis assay (Park et al, 1994).

### Change of cell cycle distribution

Cell cycle profiles of the two sensitive cell lines with time following TGF-β treatment were analysed by flow cytometric analysis. TGF-β induced G1 arrest quite rapidly, within 6 h, and by

© 1999 Cancer Research Campaign

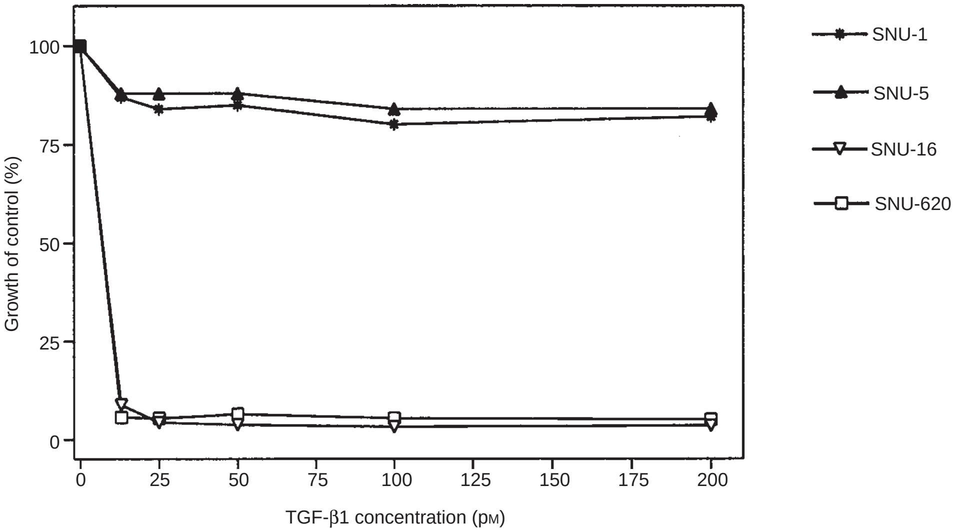

**Figure 1** TGF-β sensitivity in four gastric carcinoma cell lines. Cells ($1 \times 10^4$ cells/well) were plated in 96-well plates in the presence of porcine TGF-β1 at various concentrations. After 3 days, the MTT assay was conducted to determine relative cell numbers. Two cell lines (SNU-16 and -620) were shown to be highly sensitive to TGF-β. The assay was performed in sextuplicate

**Table 1** Changes in cell cycle profiles after TGF-β treatment in the two gastric cell lines

| | SNU-16 | | | | SNU-620 | | | |
|---|---|---|---|---|---|---|---|---|
| **Time after TGF-β treatment (h)** | **0** | **6** | **12** | **24** | **0** | **6** | **12** | **24** |
| G1 (%) | 30.1 | 38.5 | 64.1 | 85.3 | 53.5 | 59.3 | 72.4 | 86.1 |
| S (%) | 57.4 | 41.2 | 19.8 | 11.4 | 37.6 | 29.4 | 18.9 | 2.2 |
| G2/M (%) | 12.5 | 20.3 | 16.1 | 3.3 | 8.9 | 11.4 | 8.7 | 11.7 |

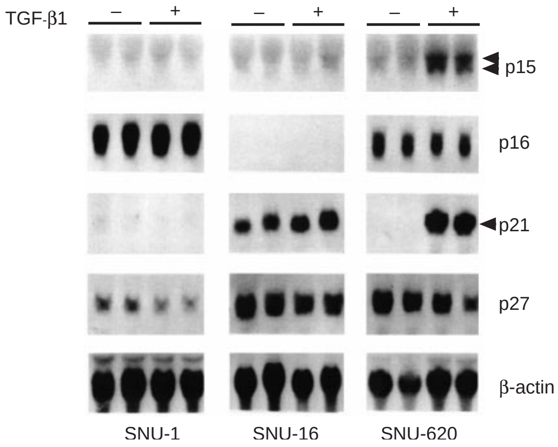

**Figure 2** Effect of TGF-β on the expression of cdk inhibitors. Northern blot analysis for several cdk inhibitors in a TGF-β-resistant (SNU-1) and two sensitive (SNU-16 and -620) cell lines. After 6 h of TGF-β treatment, total RNAs were extracted from individual cell lines cultured in two separate flasks. p21 (in SNU-16 and -620) and p15 (only in SNU-620) mRNA levels were induced by TGF-β (arrowheads indicate changes). Homozygous deletion of p15 and p16 genes caused the transcripts to be undetectable despite discernible non-specific bands in p15 panels. Expression of other cdk inhibitors did not change in any of the cell lines examined

24 h had prevented most cells from re-entering the S phase (Table 1).

### Induction of cdk inhibitors by TGF-β

Considering the fast response to TGF-β seen in cell cycle profiles, it was hypothesized that a cellular mediator would respond earlier than 6 h. In two separate experiments, RNA expression of various cdk inhibitors was examined by Northern blot analysis 6 h after TGF-β treatment. None of the inhibitors were induced in the two TGF-β-resistant gastric carcinoma cell lines (Figure 2; data not shown for SNU-5), while p21 mRNA was significantly induced in both sensitive cell lines (SNU-16 and -620). p15 mRNA levels also increased in SNU-620, but other CDK inhibitors such as p16 and p27 (Figure 2) and cdk4 (data not shown) were not affected. p15 and p16 transcription was not detectable in SNU-16, in which homozygous deletion of both genes had previously been identified (Lee et al, 1997).

### Temporal change in mRNA and protein levels of cell cycle regulatory molecules in response to TGF-β

In addition to p15 and p21, other cell cycle regulators including c-*myc* and Cdc25A have been reported to respond transcriptionally to TGF-β; all have been implicated in the early events of TGF-β-induced growth arrest. The time courses of p21, p15, c-*myc* and Cdc25A expression in both TGF-β-sensitive cell lines were therefore examined by Northern blot analysis using poly-A RNA (Figure 3). p21 mRNA levels increased within 0.5–2 h of TGF-β treatment and peaked (at ~25 times the level of controls in SNU-16 and ~52 times in SNU-620) within 6 h, whereas in SNU-620, that of p15 began to rise and reached its maximum far more slowly. C-*myc* mRNA levels were also down-regulated slightly but rapidly (within 1 h), and then sustained in both cell lines. In particular, when TGF-β was administered, Cdc25A mRNA levels were unaffected for the first 6 h, decreased and eventually completely disappeared. This does not agree, however, with previous findings

 © 1999 Cancer Research Campaign

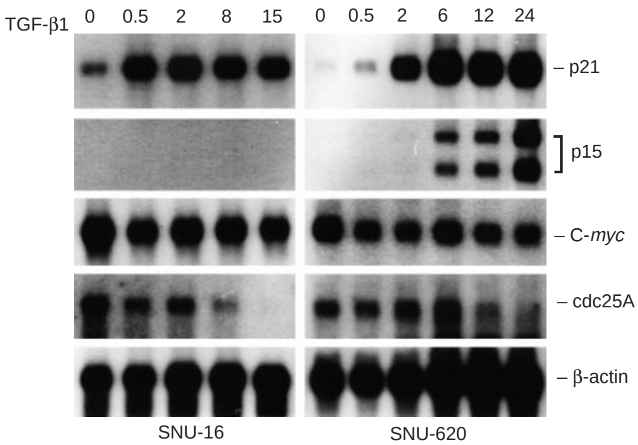

**Figure 3** Time course changes in p21, p15, c-*myc* and Cdc25A mRNA levels after TGF-β treatment in two TGF-β-sensitive gastric carcinoma cell lines. For sensitive visualization of faintly expressed transcripts such as p15, Northern blot analysis was performed using poly-A RNAs. In SNU-620, p21 mRNA level was increased more rapidly (0.5–2 h after TGF-β treatment) than that of p15, while in both sensitive cell lines down-regulation of Cdc25A mRNA occurred later (6–10 h after treatment). C-*myc* mRNA levels were decreased within 1 h by less than half and were then sustained

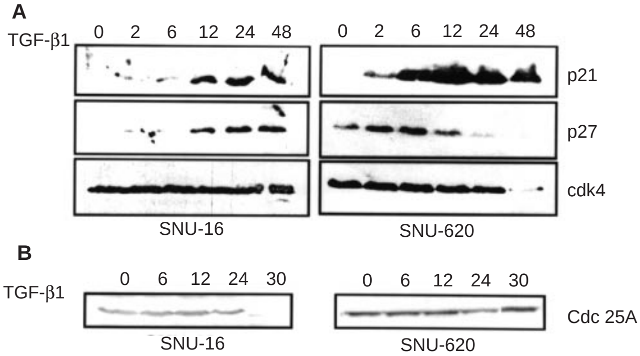

**Figure 4** (**A**) Rapid and prominent induction of p21 protein was also observed, while changes in p27 levels were different in the two TGF-β-sensitive cell lines. cdk4 level was constant until 24 h and were then sustained longer (in SNU-160) or disappeared (in SNU-620). (**B**) After 24 h, down-regulation of Cdc25A had not occurred in either cell lines, but in SNU-16 only occurred within 30 h.

which showed that in mammary epithelial tumour cells, Cdc25A repression occurred quite rapidly, which is comparable to the change of c-*myc* (Iavarone and Massague, 1997).

Rapid p21 protein induction was also observed by Western blot (Figure 4A). Although the RNA level of p27 was unaffected until 24 h (data not shown), there were changes in protein levels, with an opposite pattern in both cell lines. Within 12 h of TGF-β treatment, p27 level increased in SNU-16, but showed a marked decline in SNU-620. cdk4 levels were largely constant during cell cycle arrest, but in SNU-620, cdk4 disappeared completely after 24 h. Cdc25A protein levels did not decrease in either cell line within 24 h (Figure 4B).

### Increased p21 associates with the cdk4 complex and inhibits kinase activity

As p21 is a universal cdk inhibitor, TGF-β-induced p21 may associate with G1 cyclin kinases (e.g. cyclin D–cdk4 or cyclin E–cdk2

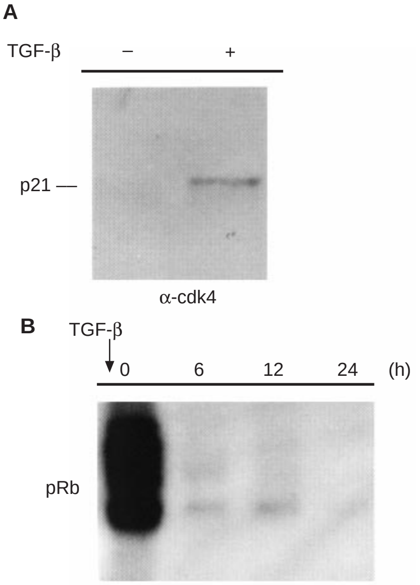

**Figure 5** TGF-β led to induced association of p21 with CDK4 and reduced kinase activity of G1 cyclin–cdk4. (**A**) After treatment with TGF-β for either 12 h or not at all, SNU-620 whole cell lysates were immunoprecipitated with anti-CDK4 antibodies and probed with p21 antibodies on Western blot. (**B**) SNU-620 cells were treated with TGF-β1 for different times and, from the lysates, the kinase activities of CDK4 immunoprecipitates were also assessed using GST-Rb as a substrate. Phosphorylation of Rb was effectively inhibited in 6 h.

complex). Anti-cdk4 immunoprecipitates were resolved on 12% SDS-polyacrylamide gel and probed with anti-p21 antibody. We observed the induced association of p21 with cdk4 in TGF-β-treated SNU-620 cells compared with untreated cells (Figure 5A), and also measured the kinase activity of the cdk4 immunoprecipitates using GST-Rb as a substrate. As shown in Figure 5B, rapidly decreasing phosphorylation of Rb was observed in TGF-β-treated cells. Because the level of p27 was not increased during that period, it is assumed that this reduced kinase activity was not attributable to this cdk inhibitor.

### DISCUSSION

G1 cdks, principally the cyclin D1–cdk4 and cyclin E–cdk2 complexes, are known to be responsible for integrating combinations of growth inhibitory signals from different sources, one of which is TGF-β receptor-generated signals, during G1 (Alexandrow and Moses, 1995). The inhibition of G1 cdk kinase activity by TGF-β was originally thought to result from suppressed G1 cyclin or cdk4 synthesis (Ewen et al, 1993; Geng and Weinberg, 1993). Later, cdk inhibitors such as p27 (Polyak et al, 1994*a*, 1994*b*), p15 (Hannon and Beach, 1994) and p21 (Datto et al, 1995; Li et al, 1995) were shown to be potent cellular mediators of the inhibitory effect of TGF-β. However, the fact that there are multiple cell cycle regulators involved in TGF-β action prompts several questions relating to their actual roles in a certain cell type, whether they act via redundancy or in a coordinate manner, and whether their relative importance is largely common or different depending on cell type.

A previous study involving two different TGF-β-sensitive cell lines (Mu1Lv mink lung epithelial cells and HaCat human keratinocytes) offered a unified model in which p15 and a Kip/Cip

*© 1999 Cancer Research Campaign* 

family molecule (either p27 or p21) cooperate to inhibit cdk function in response to TGF-β (Reynisdottir et al, 1995). In this model, an early role of TGF-β-induced p15 was emphasized, since it was shown to effectively displace p27 from cdk4 as well as suppress cdk4 kinase activity. The resulting liberated p27, which was then free to inhibit cyclin E–cdk2 (in Mv1Lu cells), or independently induced p21 (in HaCaT cells), might suppress cdk2 kinase activity.

Our results showed a different pattern of TGF-β-induced co-operation among CDK inhibitors in human gastric carcinoma cell lines. Remarkable induction of p21 was a common feature of the sensitive cell lines (SNU-16 and SNU-620) in the response to TGF-β treatment. Induction of p15 mRNA was also seen, albeit in a more delayed fashion, in SNU-620 cells. In SNU-16 cells, lack of p15 induction due to homozygous deletion suggests that p15 is not essential in TGF-β-induced cell cycle arrest. Delay or lack of p15 induction compared with p21 after treatment with TGF-β in SNU-620 and SNU-16 indicates that the proposed unifying model for the role of p15 cannot be applied to human gastric carcinoma cells. This study demonstrates that the role of individual inhibitors in TGF-β-induced growth arrest differs according to cell type (Polyak, 1996).

For both sensitive cell lines, accumulation of cells in G1 by TGF-β was quite fast and correlated with the induction of p21. In addition, increased association between p21 and the G1 cyclin–cdk4 complex was demonstrated, as well as decreased phosphorylation of Rb, which occurred within 6 h following TGF-β administration. These results suggest that p21 plays a prominent role in the arrest of cell proliferation caused by TGF-β in gastric carcinoma cell lines. Moreover, because both TGF-β-sensitive cell lines utilized in this study possess p53 mutations in both alleles (Kim et al, 1991; Park et al, 1997), p21 induction by TGF-β is likely to be p53-independent, as shown by others (Datto et al, 1995).

TGF-β treatment failed to alter mRNA levels of p27 and p16 in the TGF-β-sensitive gastric cell line studies. However, we observed a distinct change in p27 protein levels. Following TGF-β treatment, p27 levels increased in SNU-16, but decreased in SNU-620. Although the current experiment did not provide direct evidence of G1 cdk inhibition by induced p27, it is likely that p27 and p21 bind to cyclin E–cdk2 and suppress its activity in SNU-16 cells. It appears that, in this case, there is cooperation between p21 and p27, but not between p15 and a Kip/Cip inhibitor. A marked decrease in p27 protein levels in SNU-620 indicates that p27 might not play an important role in the mediation of TGF-β in the cells, even if induced p15 could displace p27 leading to a cdk2–p27 association.

The absence of changes in cdk4 protein levels for 24 h following TGF-β treatment also indicates that TGF-β-induced growth arrest is not mediated through decreased synthesis of cdk4. Delayed disappearance of cdk4 in SNU-620 cells, as shown in other cases, seems to be an adaptive event, aimed to ensure that the cells remain arrested (Reynisdottir et al, 1995).

Recently, Cdc25A, a phosphatase essential for G1–S transition, has also been suggested as a cellular mediator in TGF-β-induced growth arrest (Iavarone and Massague, 1997). Using the mammary epithelial cell line MCF10A, which lacks p15 and showed no induction of cdk inhibitors in response to TGF-β, Iavarone et al (1997) noted rapid down-regulation of Cdc25A by TGF-β at both the transcriptional and translational level, an effect comparable to the kinetics of c-*myc*. Consistent with this finding, we also observed distinct down-regulation of Cdc25A mRNA. This event occurred at a later time when compared to p15 and/or p21 mRNA induction. Changes in Cdc25A protein levels, however, were marginal, although Cdc25A protein disappeared after 30 h in SNU-16 cells. Therefore, in contrast to previous observations, down-regulation of Cdc25A seems to be less involved in the early stage of TGF-β-induced growth arrest in gastric carcinoma cells. Interestingly, a recent study suggested that p21 can compete with Cdc25A in binding to the cyclin E–cdk2 complex, and as a consequence, it inhibits Cdc25A (Saha et al, 1997). Based on these results, it can be assumed that Cdc25A could be inactivated by its competition with induced p21 in gastric carcinoma cells.

In summary, p21 appears to be the most prominent and rapid intracellular mediator of TGF-β-induced cell cycle arrest in gastric carcinoma cell lines. One additional induced CDK inhibitor, either p15 or p27, might be able to cooperate with p21. The more delayed or marginal down-regulation of cdk4 and/or Cdc25A indicates that their roles are secondary or adaptive in these cases. This study provides further evidence for cellular divergence mediating the growth-suppressive effect of TGF-β by cell cycle regulators (e.g. cdk inhibitors, cdk4 and Cdc25A). The existence of these multiple forms of cdk inhibitors may not represent simple redundancy, but may allow discrimination among different cell types in response to TGF-β.

### ACKNOWLEDGEMENTS

This work was supported, in part, by the Korea Science and Engineering Foundation (KOSEF) through the Cancer Research Center (CRC) at Seoul National University College of Medicine, by Non-directed Research Fund of Korea Research Foundation (1992) and by the HAN project of the Korean Ministry of Science and Technology (MOST 08-01-10).

### REFERENCES

Alexandorw MG and Moses HL (1995) Transforming growth factor β and cell cycle regulation. *Cancer Res* **55**: 1452–1457

Carmichael J, Degraff WG, Gazdar AF, Minna JD and Mitchell JB (1987) Evaluation of tetrazolium-based semiautomated colorimetric assay: assessment of chemosensitivity testing. *Cancer Res* **47**: 936–942

Chomczynski P and Sacchi N (1987) Single-step method of RNA isolation by acid guanidinium thiocyanate-phenol-chloroform extraction. *Anal Biochem* **162**: 156–159

Datto MB, Li Y, Panus JF, Howe DJ, Xiong Y and Wang X (1995) Transforming growth factor β induces the cyclin-dependent kinase inhibitor p21 through a p53-independent mechanism. *Proc Natl Acad Sci USA* **92**: 5545–5549

Ewen ME, Sluss HK, Whitehouse LL and Livingston DM (1993) TGF-β inhibition of CDK4 synthesis is linked to cell cycle arrest. *Cell* **74**: 1009–1020

Filmus J and Kerbel R (1993) Development of resistant mechanisms to the growth inhibitory effects of transforming growth factor-β during tumor progression. *Curr Opin Oncol* **5**: 123–129

Geng Y and Weinberg RA (1993) Transforming growth factor β effects on expression of G1 cyclins and cyclin-dependent protein kinases. *Proc Natl Acad Sci USA* **90**: 10315–10319

Hannon GJ and Beach D (1994) $p15^{INK4B}$ is a potential effector of TGF-β-induced cell cycle arrest. *Nature* **371**: 257–261

Iavarone A and Massague J (1997) Repression of the CDK activator Cdc25A and cell cycle arrest by cytokine TGF-β in cells lacking the CDK inhibitor p15. *Nature* **387**: 417–422

Kaelin WG, Pallas DC, Decaprio JA, Kaye FJ and Livingston DM (1991) Identification of cellular proteins that can interact specifically with the T/E1A-binding region of the retinoblastoma gene product. *Cell* **64**: 521–532

Kim JH, Takahashi T, Chiba I, Park JG, Birrer MJ, Roh JK, Lee HD, Kim JP, Minna JD and Gazdar AF (1991) Occurrence of p53 gene abnormalities in gastric carcinoma tumors and cell lines. *J Natl Cancer Inst* **83**: 938–943

 © 1999 Cancer Research Campaign

Lee YY, Kang SH, Seo JY, Jung CW, Lee KU, Choe KJ, Kim BK, Kim NK, Koeffler HP and Bang Y-J (1997) Alteration of $p16^{INK4A}$ and $p15^{INK4B}$ genes in gastric carcinomas. *Cancer* **80**: 1889–1896

Li C, Suardet L and Little JB (1995) Potential role of WAF1/Cip1/p21 as a mediator of TGF-β cytoinhibitory effect. *J Biol Chem* **270**: 4971–4974

Markowitz SD and Roberts AB (1996) Tumor suppressor activity of the TGF-β pathway in human cancers. *Cytokine Growth Factor Rev* **7**: 93–102

Massague J (1990) The transforming growth factor-β family. *Annu Rev Cell Biol* **6**: 597–641

Nasmyth K and Hunt T (1993) Cell cycle. Dams and sluices. *Nature* **366**: 634–635

Park JG, Frucht H, Larocca RV, Bliss DP Jr., Kurita Y, Chen T, Henslee JG, Trepel JB, Jensen RT, Johnson BE, Bang Y-J, Kim JP and Gazdar AF (1990) Characteristics of cell lines established from human gastric carcinoma. *Cancer Res* **50**: 2773–2780

Park JG, Yang HK, Kim WH, Chung JK, Kang MS, Lee JH, Oh JH, Park HS, Yeo KS, Kang SH, Song SY, Kang YK, Bang Y-J, Kim YI and Kim JP (1997) Establishment and characterization of human gastric carcinoma cell lines. *Int J Cancer* **70**: 443–449

Park K, Kim S-J, Bang Y-J, Park JG, Kim NK, Roberts AB and Sporn MB (1994) Genetic changes in the transforming growth factor β (TGF-β) type II receptor gene in human gastric cancer cells: correlation with sensitivity to growth inhibition by TGF-β. *Proc Natl Acad Sci USA* **91**: 8772–8776

Pietenpol JA, Stein RW, Moran E, Yaciuk P, Schlegel R, Lyons RM, Pittelkow MR, Munger K, Howley MR and Moses HL (1990) TGF-β1 inhibition of c-*myc* transcription and growth in keratinocytes is abrogated by viral transforming proteins with pRB binding domains. *Cell* **61**: 777–785

Polyak K (1996) Negative regulation of cell growth by TGF-β. *Biochem Biophys Acta* **1242**: 185–199

Polyak K, Kato J, Solomon MJ, Sherr CJ, Massague J, Roberts JM and Koff A (1994a) $p27^{Kip1}$, a cyclin–CDK inhibitor, links transforming growth factor-β and contact inhibition to cell cycle arrest. *Genes Dev* **8**: 9–22

Polyak K, Lee MH, Ergumnet-Bromage H, Koff A, Rorberts JM, Tempst P and Massague J (1994b) Cloning of $p27^{Kip1}$, a cyclin-dependent kinase inhibitor and a potential mediator of extracellular antimitogenic signals. *Cell* **78**: 59–66

Reynisdottir I, Polyak K, Iavarone A and Massague J (1995) Kip/Cip and Ink4 Cdk inhibitors cooperate to induce cell cycle arrest in response to TGF-β. *Genes Dev* **9**: 1831–1845

Saha P, Eichbaum Q, Silberman ED, Mayer BJ and Dutta A (1997) $p21^{CIP1}$ and Cdc25A: competition between an inhibitor and an activator of cyclin-dependent kinases. *Mol Cell Biol* **17**: 4338–4345

Wrana JL, Attisano L, Wieser R, Ventura F and Massague J (1994) Mechanism of activation of the TGF-β receptor. *Nature* **370**: 341–347

*© 1999 Cancer Research Campaign*
